# How do students reason about statistical sampling with computer simulations? An integrative review from a grounded cognition perspective

**DOI:** 10.1186/s41235-024-00561-x

**Published:** 2024-05-31

**Authors:** Sebahat Gok, Robert L. Goldstone

**Affiliations:** 1 Program in Cognitive Science, Indiana University, 1101 E. 10th Street, Bloomington, IN 47405 USA; 2 Department of Instructional Systems Technology, Indiana University, Bloomington, 201 N Rose Avenue, 47405 IN USA; 3 Department of Psychological and Brain Sciences, Indiana University, Bloomington, 1101 E. 10th Street, IN 47405 USA

**Keywords:** Statistical sampling, Dual-process theories, Grounded cognition, Statistics simulations, Perceptual learning

## Abstract

Interactive computer simulations are commonly used as pedagogical tools to support students’ statistical reasoning. This paper examines whether and how these simulations enable their intended effects. We begin by contrasting two theoretical frameworks—*dual processes* and *grounded cognition*—in the context of people’s conceptions about statistical sampling, setting the stage for the potential benefits of simulations in learning such conceptions. Then, we continue with reviewing the educational literature on statistical sampling simulations. Our review tentatively suggests benefits of the simulations for building *statistical habits of mind*. However, challenges seem to persist when more specific concepts and skills are investigated. With and without simulations, students have difficulty forming an aggregate view of data, interpreting sampling distributions, showing a process-based understanding of the law of large numbers, making statistical inferences, and context-independent reasoning. We propose that grounded cognition offers a framework for understanding these findings, highlighting the bidirectional relationship between perception and conception, perceptual design features, and guided perceptual routines for supporting students’ meaning making from simulations. Finally, we propose testable instructional strategies for using simulations in statistics education.

## Significance

Interactive computer simulations are popularly used to teach statistical sampling and inference. A substantial body of classroom-based design research has emerged over the last two decades on this topic, paralleling the interest of cognitive psychologists in statistical reasoning. This review bridges the gap by synthesizing diverse literature, from laboratory-based cognitive research to classroom-based design research, to investigate people’s reasoning about statistical sampling with interactive computer simulations. We organize the commonly occurring findings from these studies under a grounded cognition framework. Using this framework, we also identify instructional design strategies that future empirical researchers can test and statistics and data science practitioners can adopt. First, we highlight the importance of repeated exposure to simulations in a way that fosters creating perception–action routines aligned with mathematical principles. Second, we argue that intuitive representations ground students’ meaning making from simulations, and idealized representations help generalize learning. Third, we recommend that visual routines be guided during activities with simulations. Fourth, we note the separate affordances of simulations and verbal materials. Lastly, we propose that statistical processes depicted in the simulations should be reified as foundations for more advanced concepts and practices. Overall, the paper contributes to the learning theories and instructional design in the context of simulation-based learning in statistics.

## Introduction

People have strong intuitions about random sampling; these intuitions are wrong in fundamental respects; these intuitions are shared by naive subjects and trained scientists; and they are applied with unfortunate consequences in the course of scientific inquiry [...] Apparently, acquaintance with formal logic and with probability theory does not extinguish erroneous intuitions. What, then, can be done? (Tversky & Kahneman, 1971, p5–p9)

A conceptual grasp of the ideas [about statistical inference] is almost pictorial, based on picturing the sampling distribution [...] No amount of formal mathematics can replace this pictorial vision, and no amount of mathematical derivation will help most of our students see the vision. (Cobb & Moore, 1997, p. 897)

Statistical inference, defined as drawing probabilistic conclusions about a process or population based on sample data (Ben-Zvi et al., 2012), is a fundamental focus in introductory statistics education. The ability to perform statistical inference from a sample requires students to flexibly reason about core statistics concepts such as central tendency, variability, chance, and distributions. Computer-based simulations have been popularly used in introductory statistics curricula to foster a deeper, flexible, and integrated comprehension of these concepts (e.g., Lock et al., 2020; Son et al., 2021; Tintle et al., 2020). Interacting with these dynamic visual models (aka simulations), students engage in the practice of making inferences based on samples through inquiry-based investigations. For example, students may use the simulations to model data-generating processes, construct sampling distributions from random samples taken from an infinite process, chance devices, or a finite population, run randomization tests, or bootstrap samples (Pfannkuch et al., 2018). Throughout this paper, we will refer to these various simulation types as statistical sampling simulations.

While one of the core affordances of statistical sampling simulations is their effectiveness in teaching statistical inference through empirical distributions without relying on theoretical probability distributions (Rossman & Chance, 2014), our particular focus here is their perceptual and interactive affordances. Statistics education researchers have highlighted the benefits of such affordances, noting that simulations allow students to see the effects of changing input parameters (Pfannkuch et al., 2018; Ridgway, 2016) and improve students’ interpretative skills by providing quick and continuous feedback on their predictions (Carver, 2011). It has also been noted that simulations make abstract key concepts, such as chance and randomness, visible (Gehrke et al., 2021) and tap into learners’ perceptual systems whose computations are relatively effortless, automatic, and fast (Moore, 1998). In parallel, cognitive theory suggests that our perceptual systems, tuned over millions of years of evolution and accounting for a sizable portion of our brain activity, are powerful inner tools for understanding cultural innovations, such as formal scientific theories and constructs that have much briefer history (Goldstone et al., 2017).

### The current work

Given their prevalence in curricula, the following questions about statistical simulations motivate our investigation of them in the current paper. First, while the above arguments might sound compelling, it is important to identify whether empirical evidence supports the assumption that simulations are particularly beneficial for meaningful learning about statistical sampling and inference. Second, many options are available for visual representations of data and sampling processes; therefore, it is important to distinguish promising design choices from less beneficial ones to support students’ learning. Third, it is unclear how simulations are best situated within larger instructional contexts, and the chosen approach might dramatically influence learning experiences with the simulations. Pfannkuch et al. (2018) have noted that there is much to learn about how students reason through interacting with statistical models and how they integrate ideas about sample data, probabilistic models, context, and inference in technology-enhanced learning environments. To help meet this need, this review aims to investigate students’ reasoning about statistical sampling through computer simulations and identify the instructional conditions that may best support them.

The review consists of two main sections. The first section lays a groundwork through a selected literature review on how people spontaneously reason about the statistical sampling concepts targeted by the simulations. We initially interpret these findings through the lens of dual-process theories, discuss the limitations of dual-process-based pedagogies, and then introduce a grounded cognition framework as an alternative, proposing that simulations can enhance this reasoning. In the second section, adopting the grounded cognition framework, we systematically review empirical research studies that include instructional interventions with sampling simulations. Finally, we identify testable pedagogical considerations based on what we have learned through our review.

## Part A: What conceptions do people have about statistical sampling?

It is important to understand the common conceptual challenges people face when they spontaneously reason about statistical sampling so as to appreciate why educators needed to develop special interventions such as computer simulations in the first place to target these concepts. To this end, this section reviews people’s conceptions of statistical sampling through a selected body of literature. Based on the emerging patterns, we group the findings under two subsections, namely conceptions about probability and randomness and conceptions about sample size. It should be noted that this grouping does not imply mutual exclusivity. In fact, concepts across the two sections are often closely related to each other.

### Conceptions about probability and randomness

Children as young as eleven years old display some conception of sampling even without any prior instruction (Meletiou-Mavrotheris & Paparistodemou, 2015). These conceptions seem to stem from their daily life experiences and rarely comply with statistically normative notions. Children’s initial notion of a sample is that it is part of a larger and homogeneous entity, such as a cheese sample in the supermarket. This intuitive notion, however, does not transfer to conceptualizations of statistical sampling in which the entities from which samples drawn are heterogeneous, that is, display variation among the members of a population (Ben-Zvi et al., 2015). The gap between children’s conceptualization of sampling from a homogeneous and heterogeneous entity brings difficulties in their appreciation of why large random samples are needed in statistical inference. Indeed, children often mistrust simple random sampling to make reliable statistical inferences because they have no control over the sample composition with random selection (Schwartz et al., 1998) and they are concerned that it might lead to extreme outcomes that misrepresent the underlying population (Meletiou-Mavrotheris & Paparistodemou, 2015).

Schwartz et al. (1998) observed that children’s mistrust of random sampling is more prominent in cases where the outcome of interest covaries with another observed attribute. For example, in the context of surveying people’s opinions, not only the outcome of interest (opinion) but also other characteristics of the person (such as age, sex, and race) vary from one observation to another. In these cases, children have been found to prefer non-random, stratified methods to ensure they appropriately sample all combinations of traits of the population. While statisticians sometimes recommend stratified sampling, children's sampling preferences deviate from this recommendation because they prefer to select their own strata rather than representatively sampling from empirically identified strata. They try to ensure that “all kinds of people would be included” (p. 256). Thus, children show sensitivity to the notion of fairness and inclusion in the sampling process, often in statistically non-normative ways (Meletiou-Mavrotheris & Paparistodemou, 2015). A later study by de Vetten et al. (2018) suggests that adults also show similar types of distrust of simple random sampling.

Adults’ conceptions about random sampling were examined in early work in cognitive psychology. Kahneman and Tversky’s (1972) work showed that people believe that sampling outcomes should reflect the properties of the random process that generated them, that is, a random sample should look irregular. For example, in the experiment of tossing a fair coin (H = Heads, T = Tails) people judge systematic patterns in the order of certain outcomes (e.g., HTHTHTHT, TTHHTTHH) relatively less likely to occur than a specific outcome that looks more irregular (such as HHTTHTTH). The authors posited that this belief is the result of a heuristic people use to judge the probabilities of an event or sample, which they called representativeness heuristics. Under the representativeness heuristics, people determine the probability of an event or sample by the degree to which it looks similar to the essential characteristics that generated the parent population (Tversky & Kahneman, 1971). In follow-up work, Bar-Hillel (1980) extended this work by showing that people use several cues to judge the representativeness of a sample, such as the number of identical observations and sidedness (e.g., whether an observation is less than or greater than the mean). Accordingly, in the context of a normally distributed population of heights, a sample consisting of three identical or close observations is judged less likely than just about any other sample, and samples in which the data points are on both sides of the population mean are judged to be more likely than samples with all points on one side when their probabilities are equated.

Later studies found different kinds of biases besides representativeness.^1^ One is the equiprobability bias, the belief that any result of a random event is equally probable because “it is a matter of chance” (Lecoutre, 1992, p. 557). As a result of equiprobability bias, for example, people believe that getting a sum of 11 is as likely as getting 12 when two fair dice are thrown (note that 11 is twice as likely because it is a combination of two different outcomes [5,6] and [6,5]). Another one is the outcome approach, introduced by Konold (1989). People with an outcome approach use a single event, instead of a series of events, as the unit of analysis. They compute the most likely result for the single unit and then extrapolate it to a distribution of outcomes (Konold, 1989; Schwartz et al., 1998). For example, when asked about the most likely distribution of six rolls of a dice with one white and five black sides, people using the outcome approach respond with six black outcomes, contrary to what a representative heuristics approach would predict.

It may be worth noting that recent classroom studies have corroborated these biases across different age groups and cultures. More specifically, the representativeness heuristic and outcome approach have been documented among high school students, and even with undergraduate students with strong quantitative backgrounds, and preservice mathematics teachers in Ghana, Serbia, Belgium, and the USA (Heyveart et al., 2019; Hokor et al., 2021; Kaplar et al., 2021, Khazanov & Prado, 2010). Similarly, the equiprobability bias was documented in the same studies and additional ones, including fourth-grade children and high school students in Spain, South Korea, and Australia (Batenero et al., 2020; English & Watson, 2016; Park & Lee, 2019). The ubiquity of the results suggests that human biases in the reasoning of statistical sampling transcend the boundaries of psychologists’ research labs, specific countries’ borders, cultures, and decades.

### Conceptions about sample size

**Fig. 1:**
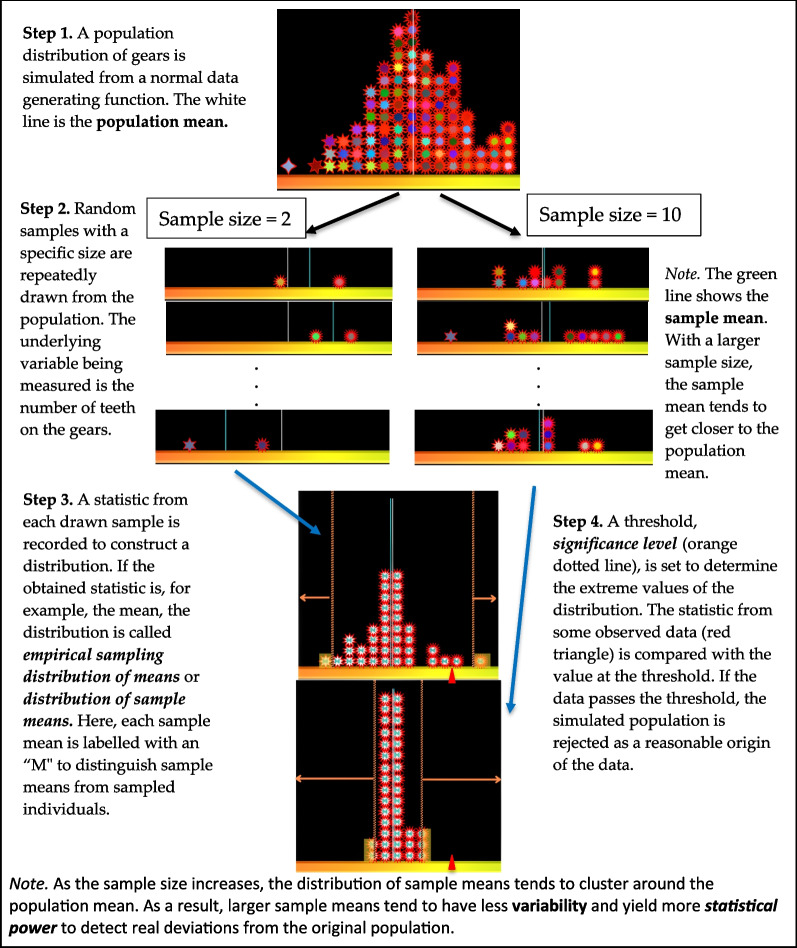
Statistical sampling

People have an intuitive sense of the law of large numbers (also called the “size-confidence intuition” by Sedlmeier, 1999). That is, people (correctly) believe that large samples generally allow for more accurate estimates of a population’s parameters than small samples. For example, when asked “A certain town is served by two hospitals. In the larger hospital, about 45 babies are born each day, and in the smaller hospital, about 15 babies are born each day. Which hospital do you think is more likely to find on one day that more than 60% percent of the babies born were boys?”,^2^ 77% of respondents were found to be able to answer the question correctly. However, then the wording of the question was converted from a single sample to a sampling distribution prompt, such that “For a period of 1 year, each hospital recorded the days on which more than 60% of the babies born were boys. Which hospital do you think recorded more such days?”,^3^ the correct responses dropped to the chance level of 33% (Sedlmeier & Gigerenzer, 1997). The results suggest that humans spontaneously appreciate the impact of sample size on the mean of an individual sample, but not on the variance of sampling distributions (see Fig. 1, Step 3).

Size-confidence intuitions seem to also disappear when reasoning about the effect of sample size on statistical power. Kahneman and Tversky (1972) found that even trained statisticians mostly judged that it was equally likely for small and large samples to have outcomes more/less extreme than a specified critical value (see Fig. 1, Note 2). Consequently, they expected that the statistical significance reached through a large sample should also be replicated with a small sample.

**Fig. 2:**
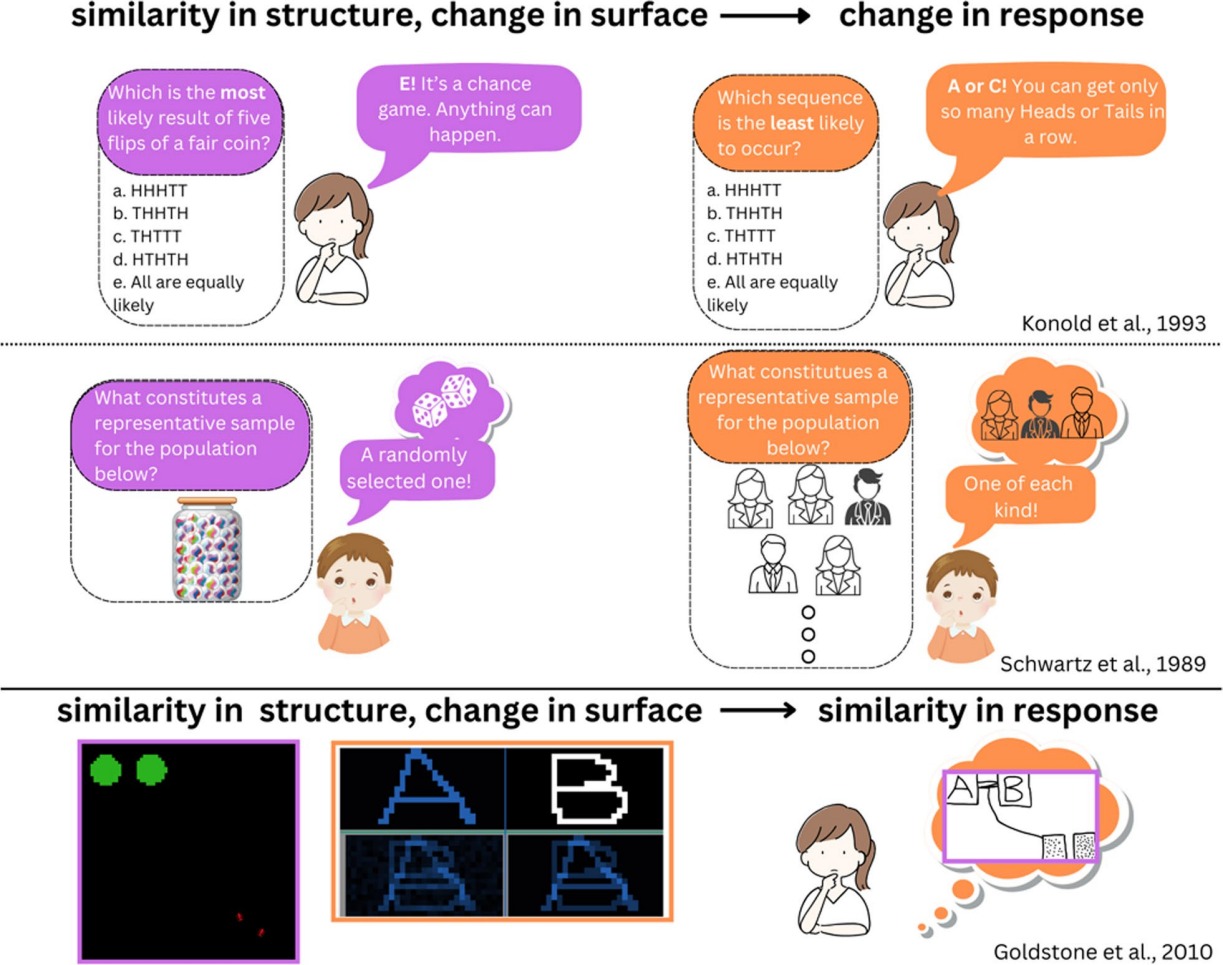
Examples of system 1 processes in reasoning. *Note.* Top and middle panels: Dual-process theories highlight the context sensitivity of people’s reasoning, which may result in inconsistent responses to structurally similar but superficially dissimilar problems—The figures are inspired by Konold et al. (1993) and Schwartz et al. (1998). Bottom panel: The proposal from grounded cognition theory is that people’s perception of contexts can be trained to be aligned with the underlying task structure—The computer simulations called *ants and food* and *pattern learning*, which are governed by the same competitive specialization principle (Goldstone et al., 2010). The sketch is redrawn from an actual participant’s sketch documented in Goldstone et al.’s study. In the bottom case, many learners successfully make the connection between the superficially dissimilar simulations by naturally interpreting the *pattern learning* situation using the same dynamic, spatial scheme that they acquired during *ants and food*. Critically, the participants are often unaware they are making the connection, indicating the intuitive and automatic nature of the process associated with System 1 thinking

To summarize, the literature indicates that people apply normative statistical rules in some situations, but they systematically deviate from them in other situations. A key question is the operational mechanisms of the heuristics that people apply: When do people, for example, choose an outcome approach over representativeness and vice versa, or use the normative statistical rules they were trained with over any heuristic? Konold et al. (1993) demonstrated that subtle changes in wording, such as asking for the most likely versus the least likely outcome in a coin flip, can lead participants to switch between an outcome approach and representativeness heuristics (see Fig. 2, Top Panel). Similarly, Schwartz et al. (1998) found that children accepted random sampling in the context of drawing marbles but not in the context of an opinion survey. The problem-solving approach that is taken seems triggered by the specific context or the framing of a problem rather than its underlying probabilistic structure (see Fig. 2, Middle Panel). These context-dependent shifts may not seem inconsistent to the participant, as each situation activates a distinct cognitive schema. However, the inconsistency becomes apparent when viewed from the underlying normative principles of probability and statistics. Schwartz et al. (1998) proposed that novices' understanding of statistics is rather fragmented and context-sensitive, drawing upon analogies with familiar scenarios akin to statistical reasoning. Consequently, the alignment of context-sensitive reasoning with normative principles depends on the extent to which the situation's representation happens to be aligned with the formal rules. The evidence reviewed also suggests that even professional statisticians can be swayed by piecemeal and context-dependent reasoning, leading them to stray from the normative principles they were trained to apply. Dual-process theories, which we introduce in the next part, can account for this piecemeal and context-sensitive reasoning phenomenon through the general structure of human cognitive architecture.

### Dual-process accounts of reasoning about statistical sampling

Dual-process theories postulate that humans possess two separate learning mechanisms. The first is an associative mechanism produced by neural networks, and the second is a rule-based system that involves the manipulation of internal symbolic structures (McLeod et al., 1998). Dual-process accounts of cognition postulate that humans have evolved a slow, deliberate, rule-based, and domain-general reasoning system (System 2) which co-exists with an older, autonomous, fast, intuitive, and associative set of sub-systems (System 1) (Evans, 2003, 2008, 2012; Kahneman & Frederick, 2005; Sloman, 1996). According to these accounts, System 1 encodes the underlying statistical structure of the environment and executes computations based on the current task’s similarity to the prior experiences, whereas System 2 learns without reliance on situation-specific experience. Logical, causal-mechanical reasoning and abstract hypothetical thinking are attributed to System 2, which is postulated to be uniquely human and evolutionarily more recent system.

According to Kahneman and Frederick (2005), when a statistical sampling problem is embedded in a verbal scenario, the contextual features of the scenario trigger objects to be mentally represented. System 1 operates on these objects in a similar fashion to how perceptual systems operate on real objects in an automatic and parallel manner. System 2 monitors the quality of System 1’s representations and may endorse, correct, inhibit, or override them. If both systems fail, errors and biases will occur.

Kahneman and Frederick (2005) propose that a computationally difficult question is often unconsciously substituted with a perceptually similar and simpler analog, which they called, the *attribute-substitution model of heuristic judgment*. For example, when asked, “If a sphere were dropped into an open cube such that it just fits, what proportion of the volume of the cube would the sphere occupy?” (p. 270), people respond as if they were asked “If a circle were drawn inside a square, what proportion of the area of the square does the circle occupy?” (p. 270). In this case, the target attribute in the judgment (that is, the volumetric relationship between the cube and sphere) is replaced by the heuristic attribute (that is, the ratio between areas of the circle and the square), a relevant perceptual impression that allows simpler computation. Critically, the respondents are not aware of having made the substitution.

The attribute-substitution model of heuristic judgment can also explain why people’s intuition of the law of large numbers vanishes in the context of computationally difficult problems such as the sampling distribution of statistics.^4^ For example, Sedlmeier and Gigerenzer (1997) found that when asked to construct sampling distribution graphs, adult subjects’ drawings were indistinguishable from a single sample graph. In another study, Well et al. (1990) found that when participants were given tasks about distribution of sample statistics (see Fig. 1, Step 3), they recalled the contents of the task as an individual sample task. Furthermore, errors in estimating variability in sampling distributions (see Fig. 1, Note 2) seem to be resistant to training (Chance et al., 2004; van Dijke-Droogers et al., 2021a). The attribute-substitution model of heuristic judgment can account for the results from these findings—the respondents replace the target attribute (distribution of sample statistics), which is difficult to calculate, with the heuristic attribute (distribution of a single sample), without being aware of this substitution.^5^

#### Pedagogical implications of dual-process theories and their limitations

Nonnormative biases in sampling tasks can be reduced or eliminated by making the statistical nature of the task explicit so that the corrective functions of System 2 processes are evoked (Kahneman & Frederick, 2005). Some empirically successful examples of this approach include asking participants to think like statisticians (Schwarz et al., 1991), drawing samples from an urn to emphasize randomness (Sedlmeier, 1999), or increasing the accessibility of rules (Macchi, 1995; Stanovich & West, 2002). Another way is stating the questions with relative frequency formats (e.g., 1 in 10) instead of their equivalent probabilities and percentages (e.g., 0.10, 10%) (Cosmides & Tooby, 1996; Gigerenzer & Hoffrage, 1995).^6^

The broader literature on scientific and mathematical expertise corroborates the idea that System 2’s corrective operations have a critical role in reaching normative solutions. As humans develop expertise in science and mathematics, they show an activation shift from posterior brain areas associated with perceptual processing to inhibitory frontal areas (more specifically, dorsal lateral prefrontal cortex and anterior cingulate cortex) (Ferrer et al., 2009; Houdé & Borst, 2014; Mareschal, 2016). This neuroimaging evidence has resulted in pedagogical approaches that train students’ System 2 to inhibit System 1’s automatic responses. One such approach is “prefrontal pedagogy” in which students are taught to take a moment of waiting time before responding (Houdé & Borst, 2014). Another is teaching students to “privilege science over intuition” (Shtulman & Legare, 2020). These pedagogical interventions have been found to improve reasoning in some science classes (for reviews, see, Houdé & Borst, 2014; Shtulman & Legare, 2020).

A word of caution is that the promises of System 2 training for statistics education may be more limited in scope than it is for other scientific disciplines. Modern statistics curricula eventually aim for students to be able to build statistical “*habits of mind”* (Ridgway, 2022, p. 3) in civic context for productive involvement in civil society, that is, spontaneously bring statistical knowledge into mind when they encounter data claims about the economy, migration, health, wealth, and the environment (Engel, 2017; Ridgway, 2016). When confronted in daily life, we may assume that these contexts will often not include explicit cues that will point out the statistical nature of the information, so there is the risk that people will be more susceptible to non-statistical reasoning when thinking about these societal problems. Indeed, research indicates that students do not transfer statistical reasoning skills to social events (Meletiou-Mavrotheris, 2007). The probabilistic nature of social events is not as explicit to students; therefore, their prior beliefs and contextual knowledge dominate their reasoning, which can result in deterministic judgments and prejudices.

Therefore, it is essential that learners develop the ability to perceive future situations through the lens of the principles and concepts they acquired during their formal education. This necessitates shifting the focus from the distinctions between System 1 and System 2 toward the interplay between the systems and, in fact, dissolving the sharp division between the two. For the remainder of this article, we will claim that this perspective is not only pedagogically optimistic but also psychologically plausible. The next section introduces this perspective under the grounded cognition framework. Based on this framework, we will argue that perceptually grounded pedagogies, such as interactive computer simulations, hold the potential for learners to form mental models that spontaneously enable new ways of viewing and understanding situations (see Fig. 2, bottom panel). This perspective also aligns with psychological theories such as *ecological rationality*, which emphasize the importance of providing individuals with appropriate representations and learning experiences to foster sound statistical intuitions (Gigerenzer, 2023), and *conceptual ecology* in education, highlighting the continuum from early intuitive understanding to more advanced stages of understanding (diSessa, 2002).

### Introduction to the grounded cognition perspective and its pedagogical promises

Much of thinking turns out to be seeing if seeing is properly understood. (Kellman & Massey, 2013, p. 120).

As opposed to the standard separation drawn between lower-level (perceptual, implicit, associative) and higher-level (abstract, logical, rule-based) cognitive processes, the grounded cognition perspective proposes that perception, action, environment, and amodal symbols all work together to create cognition (Barsalou, 2008). This perspective suggests a reciprocal tuning between perception and abstract rule-based reasoning, in which rule-based reasoning often has perceptual origins and perceptual systems contain mechanisms typically associated with abstract cognition (Goldstone & Barsalou, 1998). Converging with this notion, the later evidence from dual-process literature suggests that intuitive systems are capable of reaching normative probabilistic and logical problem solutions—an ability typically attributed to System 2. This is evidenced by the respondents’ nonverbal cues indicating doubt and conflict when verbalizing normatively incorrect responses (Bago & de Neys, 2017; Gangemi et al., 2015; Simon et al., 2015). Furthermore, experts’ successful decision-making often relies on intuitive rather than reflective thinking, and incorrect deliberate reasoning can sometimes regrettably override correct intuitions (for reviews, see, Evans 2008; 2012).

Highly skilled performance is often achieved by transforming effortful System 2 activities to effortless System 1 activities (Kahneman & Frederick, 2005). For example, rather than explicitly recalling and deploying algebraic order of precedence rules to correctly calculate 2 + 3 X 5 as 17 rather than 25, people often solve math problems by developing automatic attentional routines to attend the “X” operator before “+” (Landy & Goldstone, 2007). With time and practice, associative and perceptual processes come to be able to reach the same correct solutions as those achieved by deliberate and sequential processes (Sloman, 1996). One advantage of converting rule-based processes into improved routines for perceiving and attending to a situation is that cognitively expensive executive resources are thereby freed up and available for other aspects of a problem that have not yet become fluently processed (Zelazo, 2015).

The grounded cognition perspective is reminiscent of the distributed cognition perspective (Clark, 2017; Hutchins, 2000), in which cognition is extended to include the tools that we as humans recruit, building cognitive systems that include us as just one component. However, in the case of converting from System 2 to System 1 processes, the cognition is still being done inside one person’s brain, so instead of talking about off-loading processes from the brain onto the world, as we do when calculators and calendar reminders take over tasks that we once did ourselves, we could talk about in-loading, in which tasks are taken over by automated devices that we create inside our own brains (Goldstone, 2019). While these devices, once created, can be deployed quickly and without requiring executive resources, training them typically requires System 2 resources. In fact, executive function resources play a crucial role in acquiring what will become automatic and fluent skills in reading (Altemeier et al., 2006) and math (Purpura et al., 2017). One of the primary functions of System 2 is to make itself unnecessary by training System 1 to do the right thing according to System 2’s rules (Goldstone et al., 2015).

Understanding cognition as grounded in perception and action has informed pedagogical practices in science and mathematics. Broadly, this line of research has focused on the links between representations that require sensory encoding and abstract, formalized symbol systems (for reviews, see Nathan, 2020; Weisberg & Newcombe, 2017). More specifically, the studies showed that sensorimotor actions support insights into abstract concepts in geometry (Nathan & Walkington, 2017) and science (Hayes & Kraemer, 2017), and help coordinate multiple concepts in statistics (Son et al., 2017). The studies also have shown that top-down processes guide perceptual routines, which, in turn, influence graph interpretation (Michal & Franconeri, 2017). Computer-based simulations, anchoring visuospatial and dynamic aspects of the scientific and mathematical principles, have been shown to help students acquire the ability to perceive future analogous situations in light of their sensory and bodily experiences with the simulations (Day & Goldstone, 2011; Goldstone & Wilensky, 2008). Importantly, this influence seems to operate below the participants’ explicit awareness, suggesting the System 1-like nature of the mental models grounded in spatial and dynamic representations.

In an independent vein of research, statistics educators over the past two decades have been developing computer-based simulation pedagogies. Different from the simulation studies grounded in psychological theories, this direction is largely driven by the educational challenges encountered in teaching and learning statistics. The aims of these pedagogies include clarifying the core logic of inference, enhancing statistical intuition (Tintle et al., 2015), providing simpler-to-grasp models through randomization tests (Cobb, 2007), enabling an understanding of probability calculations without relying on complex mathematics (Bargagliotti et al., 2020; Rossman & Chance, 2014), and a concrete demonstration of sampling variability (Bargagliotti et al., 2020). To provide reciprocal understanding in both domains, this paper synthesizes the grounded cognition framework and simulation-based instruction in statistics education research. In the following section, we conduct a systematic review of statistics education literature focusing on sampling simulations through the lens of grounded cognition.

## Part B: How do students reason about statistical sampling with computer simulations?

In the previous section, we discussed the canonical dual-process theories, which suggest that humans often rely on intuitive, associative, and perceptual processes that are often misleading when reasoning about statistical sampling. We proposed an alternative view: Perceptual systems can productively be involved in abstract, conceptual, and rule-based reasoning. Cultural tools such as computer simulations may enable us to perceive forms that evolution did not initially equip us to process.

In the current section, we review the educational literature that focuses on improving students’ reasoning about statistical sampling and inference through interactive computer simulations. In these studies, a classroom is typically exposed to a particular treatment that involves interacting with simulations in a statistics course, and students’ learning is assessed during or after the intervention.

### Literature search

We conducted a systematic literature search in relevant research databases (last update: December 24th, 2022). First, we used the databases of the Web of Science and ERIC Education Resources Information Center. We initially used the following search terms (“sampling” AND “simulation”) on Web of Science (initial hit number: 49,323), which we limited by filtering the topic to “Education and Educational Research.” On ERIC, we added the keyword “AND education” for the return of more educationally relevant hits (initial hit number: 127). We then expanded our search in Google Scholar (initial hit number: 589,000). We used the following inclusion criteria:

1. Articles written in the last two decades (2002–2022).
2. Articles reporting empirical studies with computer simulations that teach statistical sampling.
3. Peer-reviewed papers (journal articles or conference proceedings).
4. Articles written in English.

We reviewed the titles and abstracts of all papers in Web of Science and ERIC. Additionally, we sampled the first 385 papers that were ordered by their relevance in Google Scholar, following the literature sampling suggestions by Onwuegbuzie and Frels (2016). As a result of this process, 20 papers were identified. From the reading of referenced work in these papers, we identified 13 more papers that met the inclusion criteria. Thus, in total, we included 33 unique papers in our review.

We did not impose any constraints on the inclusion criteria based on the empirical methods the studies used (see Table 1). As a result, 28 studies were either pre-post-test comparisons within a single group or observational qualitative studies that probed students’ understanding 4 other studies included an additional no-simulation comparison group. A single study used a quasi-experimental design to compare two simulation activities. Notably, none of the studies included controlled experiments.

**Table 1:** The summary of the systematically reviewed studies

Authors and date	The software	Method(s)	*n*	Setting	Instructional activities	Learning measures	Positive learning outcomes	Conceptual challenges
Abrahamson et al. (2006)	Netlogo	Design-based research	40	6th graders, the USA	Inquiry activities	Post-test, interviews	Conception of signal and noise	Context-dependent reasoning
Arnold et al. (2017)	A dynamic boxplot simulation	Design-based research	40	10th graders, New Zealand	Inquiry activities Use of physical devices	Field observations Teacher reflections Pre–post-test Interviews	General conceptual understanding	Inference from a single sample
Bakker (2004)	A histogram simulation	Design-based research	30	8th graders, Netherlands	Growing a sample Whole-class discussion	Student drawings Field observations Think-aloud interviews	Aggregate view of data	NA
Ben-zvi et al. (2012)	Tinkerplots	Design-based research	5	5th graders, Israel	Inquiry activities Growing a sample	Interviews	General use of probabilistic language	Missing global patterns by overfocusing on local variability
Braham et al. (2013)	Tinkerplots	Design-based research	2	7th graders, Israel	Inquiry activities	Interviews Field observations	NA	Inference from a single sample *n* versus *n*/*N*
Chandrakantha (2014)	Excel	Comparative study between simulation versus no-simulation instruction	48	Undergraduate students, the USA	Inquiry activities	Final exams	Overall performance improvement	NA
Chandrakantha (2018)	R	Comparative study between simulation versus no-simulation instruction	36	Undergraduate students, the USA	Inquiry activities	Final exams	Overall performance improvement	NA
de Vetten et al. (2018)	Vustat	Design-based research	21	Preservice teachers, the Netherlands	Inquiry activities Lecture Demonstration	Field observations Pre-post-test	Relationship between variability in sampling distributions and sample size	*n* versus *n*/*N* Inference from a single sample
Eliason and Jones (2020)	Rice Virtual Lab	Interview study	5	Preservice teachers, the USA	NA	Interviews	NA	Inference from a single sample
Findley and Lyford (2019)	NA	Case study	8	Undergraduate students, the USA	NA	Interviews	NA	Relationship between variability in sampling distributions and sample size Context-dependent reasoning
Hancock and Rummerfield (2020)	Rossman and Chance applet	A quasi-experiment that compares computer-based simulation versus hands-on activities + computer-based simulation	386	Undergraduate students, the USA	Inquiry activities Use of physical devices	Exam scores Reflection reports	General conceptual understanding	NA
Jacob and Doerr (2014)	Fathom	Design-based research	14	11–12th graders, the USA	NA	Interviews	Sampling distributions as a distribution of sample statistics Constructing-confidence intervals Relationship between variability in sampling distributions and sample size	Interpreting p-values Interpreting confidence intervals Inference from a single sample
Konold and Kazak (2008)	Tinkerplots	Design-based research	28	7–8th graders, the USA	Inquiry activities Use of physical devices	Interviews	Conception of signal and noise	Missing global patterns by overfocusing on local variability Compound probabilities Relationship between variability in sampling distributions and sample size
Lehrer et al. (2014)	Tinkerplots	Design-based research	13	6th graders, the USA	Inquiry activities	Interviews	Understanding sampling distributions as a distribution of sample statistics	Relationship between variability in sampling distributions and sample size
Lehrer (2017)	Tinkerplots	Design-based research	12	6th graders, the USA	Inquiry activities	Interviews	Conception of signal and noise Expected value of mean	NA
Lunsford et al. (2017)	SamplingSIM	Design-based research	25	Undergraduate students, the USA	Inquiry activities Demonstration	Pre-post-test	NA	Averaging reduces variability Sample mean as a random variable
Makar et al. (2011)	Tinkerplots	Design-based research	3	6th grade (aged 11–12), Israel	Inquiry activities	Interviews	General display of statistical norms and habits	NA
Maxara and Biehler (2006)	Fathom	Case study	13	Undergraduate Mathematics Education students	NA	Interviews	Successful analogical reasoning after being cued	*n* versus *n*/*N* Context-dependent reasoning
McDaniel and Green (2012)	Rossman and Chance applet	Design-based research	177	Undergraduate and graduate students, the USA	Inquiry activities	Pre-post-test	General conceptual understanding	NA
McLean and Doerr (2015)	Tinkerplots	Design-based research	4	Secondary and tertiary level students	Inquiry activities Use of physical devices	Interviews Field observations	General display of statistical norms and habits	Bootstrap resampling
Meletiou-Mavrotheris and Paparistodemou (2015)	Tinkerplots	Design-based research	19	4th–6th graders, Cyprus	Inquiry activities	Interviews	Meaning and role of sample, randomization, and sample size	*n* versus *n*/*N*
Pfannkuch et al. (2015)	A dynamic boxplot simulation	Design-based research	21	15-year-olds, New Zealand	Inquiry activities	Pre-post-tests Interviews Field observations	Relationship of variability in sampling distributions and sample size Inference from a single sample	Relationship of variability in sampling distributions and sample size *n* versus *n*/*N*
Pratt et al. (2008)	Chancemaker	Design-based research	4	10–11 years old	Inquiry activities	Interviews	NA	Missing global patterns by overfocusing on local variability
Saldanha and Thompson (2002)	A histogram simulation	Design-based research	27	11–12th grade, the USA	Inquiry activities	Field observations Think-aloud interviews	NA	Inference from a single sample
Saldanha and Thompson (2007)	Prob Sim	Design-based research	8	10–11th grade, the USA	Inquiry activities	Interviews Field observations	Sampling distributions as a distribution of sample statistics	NA
Saldanha (2016)	Prob Sim	Design-based research	8	High school students, the USA	Inquiry activities	Field observations	NA	Quantifying expectations Imagining situations as stochastic experiments
Salinas-Herrera and Salinas-Hernández, (2022)	Fathom	Design-based research	18	17–18 year-old, high school students	NA	Field observations Teacher reflections Think-aloud interviews	Aggregate view of data	Normal distribution as an approximation to binomial distribution
Smith (2004)	Smith's Statistical Package	Design-based research	30	Undergraduate students in introductory statistics course, the USA	Inquiry activities	Pre-post questionnaire	*n* versus *n*/*N*	NA
van Dijke-Droogers et al. (2021a)	Tinkerplots	Design-based research	27	9th graders, the Netherlands	Inquiry activities	Think-aloud interviews Field observations	General conceptual understanding	NA
van Dijke-Droogers et al. (2021b)	Tinkerplots	Comparative design-based research	217	9th grade (14–15 years old), the Netherlands	Inquiry activities Use of physical devices	Pre-post-test Student worksheets Teacher notes Field notes	General display of statistical norms and habits General conceptual understanding	Relationship between variability in sampling distributions and sample size Inference from a single sample
Vanhoof et al. (2007)	A histogram simulation	Design-based research	221	Undergraduate students, Belgium	Practice with simulations followed by lecture	Pre-post-test	Sampling distributions as a distribution of sample statistics	Intuitive law of large numbers for non-graphical transfer items
Vaughn (2009)	R	Quasi experiment	244	Undergraduate students, the USA	Demonstration. practice	Pre-post-test	Overall understanding of central limit theorem	NA
Watkins et al. (2014)	A histogram simulation	Teacher PD	9	High school teachers	Practice with simulations	Interviews	Relationship between variability in sampling distributions and sample size	Expected value of mean Expected value of standard error

### Information retrieval

For each paper, we coded information about authors, publication date, the simulation software that was used, research methods, sample size, setting, instructional activities that simulations were situated in, learning measures, and learning outcomes. (We included outcomes from both qualitative and quantitative measures.) We sorted learning outcomes according to two dimensions: positive learning outcomes and conceptual challenges (see Table 1).

### Findings

We report the themes that consistently appeared across multiple studies (see Table 1, Columns “Positive learning outcomes” and “Conceptual challenges”). For each theme, we first summarize the studies, followed by details we view as important regarding simulation design and learning activities. We conclude each section with our overall interpretation of the evidence.

#### Benefits of simulations for general statistical reasoning abilities and skills

Several studies reported positive learning outcomes in relation to general statistical reasoning abilities and skills. The reported evidence was better performance in items that target general statistical reasoning when compared to non-simulation groups (Chandrakantha, 2014, 2018; van Dijke-Droogers et al., 2021a), improvement from pre-to-post-test (Arnold et al., 2017, Konold & Kazak, 2008; Lehrer, 2017; McDaniel & Green, 2012); and qualitative observations of increased adoption of statistical norms, habits, and the use of probabilistic language (Braham et al., 2013; Makar et al., 2011; McLean & Doerr, 2015; van Dijke-Droogers et al., 2021b).

An important learning objective in basic statistical education is developing habits of mind, defined as the ability to spontaneously bring statistical knowledge to bear when one encounters critical claims about data (Ridgway, 2022). Sampling simulations have been found to help learners develop appropriate statistical norms and habits (Makar et al., 2011; McLean & Doerr, 2015; van Dijke-Droogers et al., 2021b). Rather than the specific simulation software per se, the studies collectively emphasize the role of the accompanying pedagogical activities. In one study, Makar et al. (2011) attributed benefits to the inquiry activities, discussion, checklists, and continued exposure to data through simulations. They found that checklists that accompany data investigations directed students’ attention to the centers, spreads, and outliers of graphs, which allowed them to view the center as a meaningful representation of the group while also considering the role of variability. Students developed more sophisticated inferences across repeated trials which improved their conceptual sophistication. In another study, van Dijke-Droogers et al. (2021b) found that compared to the comparison group, students who were taught with simulations more often drew conclusions based on data with reference to statistical information and probabilistic reasoning and less often based on personal intuition and bias. The authors attributed the outcomes to the inquiry activities in which Tinkerplot simulations were embedded.

An important feature of simulations is their affordance for allowing observation of sampling variability over time. In a study with sampling simulations, Ben-Zvi et al. (2012) found that students at the beginning of instruction tended to be extreme in their interpretations of data. That is, they were either claiming to know something for sure or that nothing could be inferred from the data. However, over time, with engagement with simulations, they increasingly saw evidence for or against particular statements, which resulted in them developing a probabilistic language to specify their level of confidence, such as “the chances are … really small” or “it seems that…”. With prompting by researchers, they further quantified such confidence levels. Similarly, Konold and Kazak (2008) and Lehrer (2017) observed that, through multiple repetitions, students develop a better sense of sampling variability by observing what remains similar from sample to sample. Thus, students co-develop perception and conception of the idea of data as consisting of signal and noise as they explore data through multiple iterations.

**Fig. 3:**
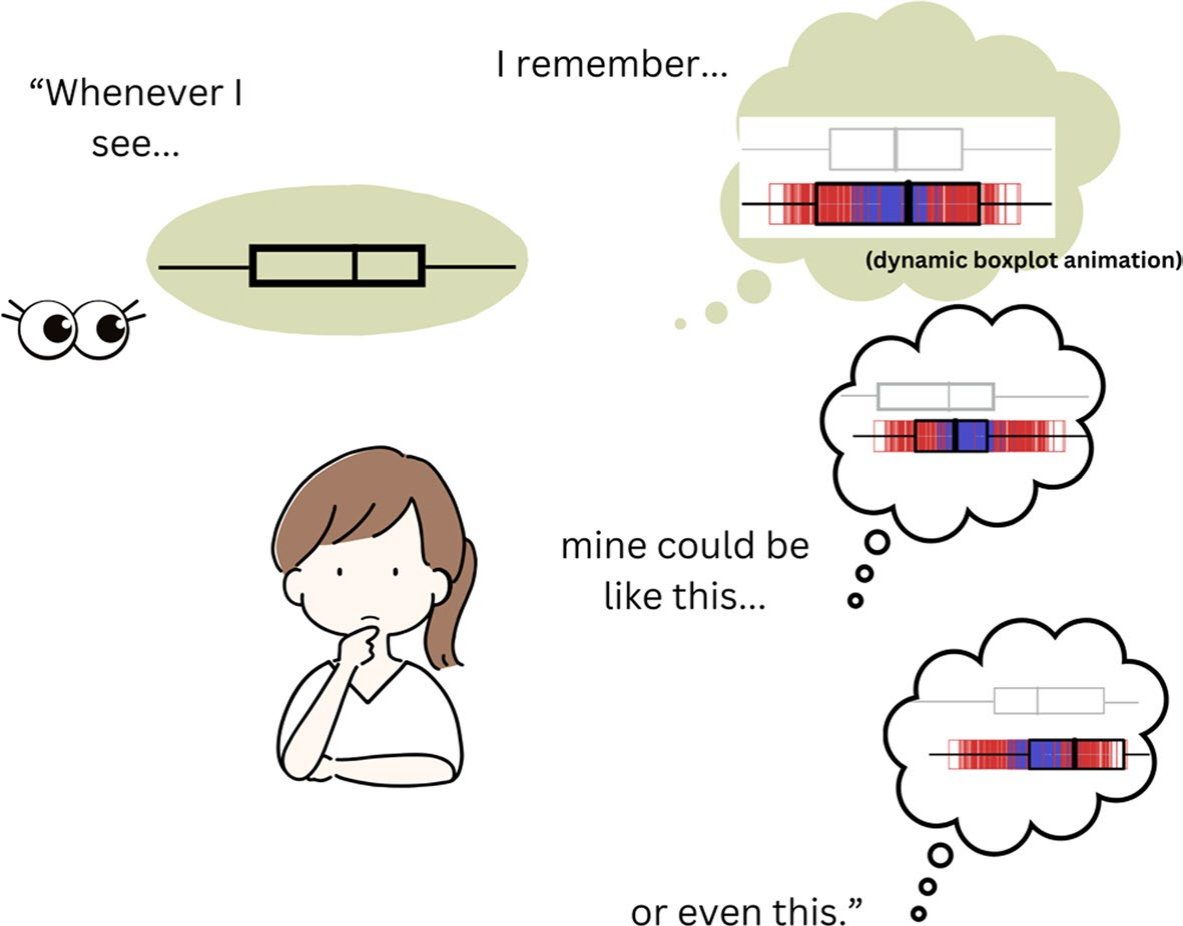
Building habits of mind. *Note.* The figure illustrates how sampling simulations can cultivate habits of mind. Here, by recalling the dynamic boxplot animation that models sampling distribution, the student considers the uncertainty resulting from the sampling variability when they view a single sample. The figure was redrawn from Arnold et al. (2017). The colorful boxplot is a screenshot from https://www.stat.auckland.ac.nz/~wild/WPRH/

To summarize, previous research suggests that sampling simulations can improve informal statistical inference skills by developing *habits of mind* and improving conceptual understanding of sampling variability over repeated trials (see Fig. 3). Inquiry activities accompanying the simulations have been found helpful in eliciting such benefits, with a few features specifically highlighted. First, continued exposure to data results in increasingly more sophisticated interpretations. Second, observation of central tendency and variability across samples helps learners to develop interpretations of data as *signal and noise*. Third, guidance of inquiry through *interpretive checklists* seems to be a potentially effective way of focusing students’ exploration of important aspects of graphs.

#### Local versus global view of data

Studies conducted with elementary and middle school children show that students have a case-oriented view (such as focusing on individual data points or single frequencies) rather than an aggregate view (such as focusing on an aggregation of data, relative frequencies, or overall distribution shape) (Ben-Zvi et al., 2012; Konold & Kazak, 2008; Pratt et al., 2008). For example, a student might pay attention to the minor deviations between consecutive samples taken from the same population, missing the important invariant that the means of those samples are very similar. In simulation activities, this local emphasis makes it difficult for students to appreciate that large samples are overall better estimators than small samples because they observe that there is still some variability from one large sample to another. In other words, the invariance of statistics such as the mean is overridden by the tendency to perceive local change (Konold & Kazak, 2008). This perceptual bias was consistent across different types of diagrams, namely pie charts, pictogram bars, and histograms (Ben-Zvi et al., 2012; Konold & Kazak, 2008; Pratt et al., 2008).

In one study (Pratt et al., 2008), after investigating simulated data, middle school children concluded that taking large samples was inappropriate because even large samples did not perfectly match the population. In a similar study setup, Konold and Kazak (2008) found that students judged smaller samples to be better estimators, focusing on the likelihood of getting a perfect match to the expected value rather than the overall error around the expected value. In these cases, the focus on local changes in large sample simulations seems to have overridden students’ size-confidence intuition and invoked the growing possibilities heuristic instead. Growing possibilities refer to the belief that more opportunities exist to deviate from the population parameters with larger samples as there are more unique observations (Findley & Lyford, 2019). By this line of reasoning, children believe that every data point in a large sample could deviate substantially from the population mean, and if even one data point is off, then the sample distribution is invalid.

**Fig. 4:**
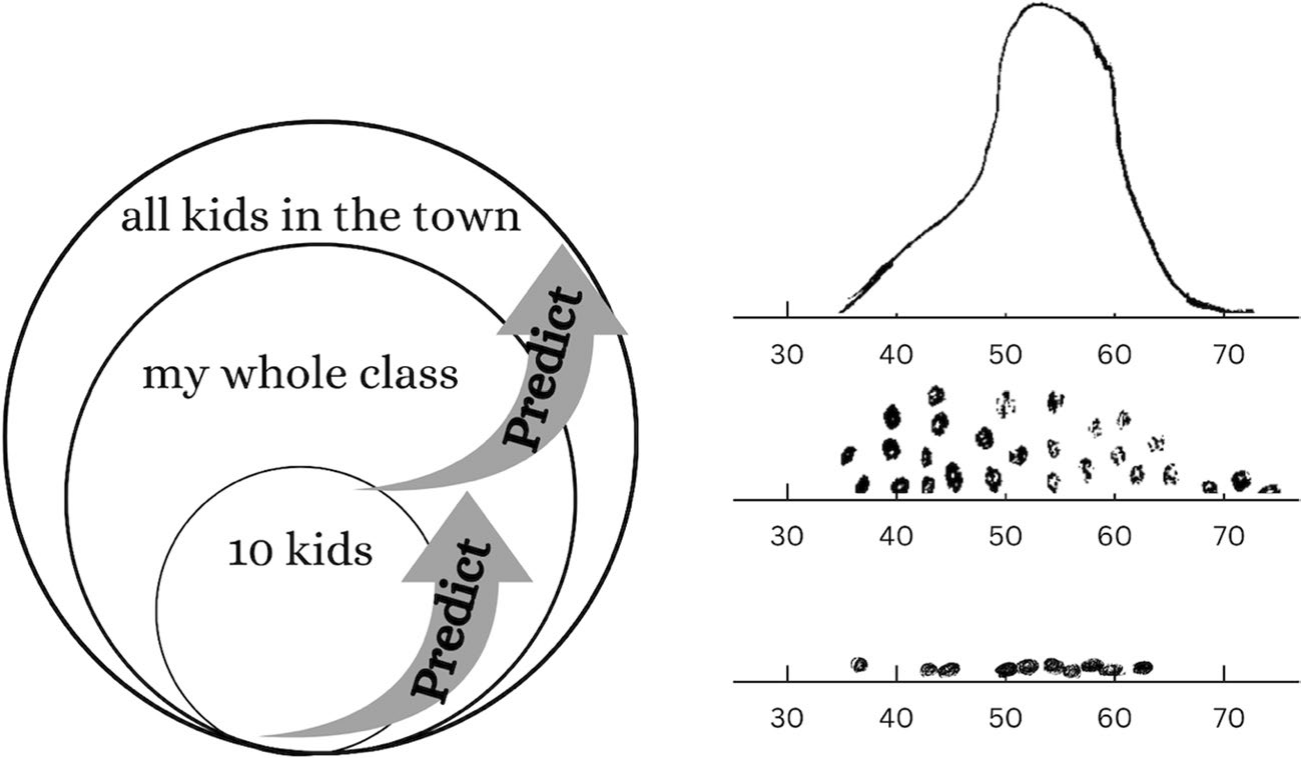
Growing a sample activity. *Note.* The progression of the activity involves students increasingly drawing and observing larger data sets. The goal of the activity is to gradually shift from a case-oriented to a distributional view of the data. The figures are redrawn and adapted from Ben-zvi et al. (2012) and Bakker (2004)

To alleviate the problem of overfocusing on the local properties of data at the expense of the global view, Bakker (2004) devised a “growing a sample” activity to improve students’ aggregate perspective on data. In this activity, middle school students graphed their prediction of children's weights with varying sample sizes—from 10 children to a class, followed by three classes, and finally, the city’s entire child population. In the initial phases of the activity, students created simple dot plots to represent individual weights, which transitioned into graphs with continuous shapes, such as histograms or density plots, in the final stage representing the entire population. After each prediction phase, the students would compare their graphs with those of actual data samples of equivalent size provided by their teacher. The author designed this activity to gradually shift the students’ attention from the individual data points to the overall data distribution (see Fig. 4).

This activity was designed to foster a distributional rather than a case-oriented view of the data. Bakker observed a noteworthy linguistic transition in student discussions during this process: during the initial cycle with the smallest sample size, students used adjectival predicates such as “together,” “apart,” and “spread out” to refer to trends in data (e.g., “The dots are more spread out”); however, as cycles progressed, they tended to replace these terms with nouns such as “spread” and “average.” (e.g., The spread is larger), which suggests that students’ language use also became aggregate-oriented.

The author argues that such a linguistic shift is crucial for forming object-like concepts for statistical reasoning so that these objects can become something that students can reason *with*. However, it is important to note that Bakker’s findings, drawn from qualitative observation of class activities, do not assert a causal, or even correlational, relationship between this linguistic shift and a more developed aggregate view. Indeed, Bakker himself noted, “Of course, this does not always imply that if students use these nouns that they are thinking of the right concept (p. 73)”.

Nevertheless, it is well-documented elsewhere that perceptual chunks provide a basis for experts’ problem-solving (Chase & Simon, 1973; Koedinger & Anderson, 1990). From this perspective, transformations from predicates to nouns may reflect a broader learning strategy in which information is packaged into larger chunks to form and process higher-level units. Generative activities that guide such transitions, whether visual (e.g., from dot plots to histograms) or verbal (e.g., from predicates to nouns), can ground aggregate views on data for grasping richly structured concepts.

#### Conceptions about the variability of the sampling distributions

Several studies indicate that students often fail to appreciate that variability decreases in sampling distributions as the sample size gets larger even after they observe it in the simulated distributions (Findley & Lyford, 2019; Konold & Kazak, 2008; Lehrer et al., 2014, van Dijke-Droogers et al., 2021b). Students continue to believe the variability in the sampling distribution of means does not change with the sample size, or that it gets larger with increases in sample size. An important problem seems to be that students fail to grasp that the sampling distribution is a distribution of sample statistics, not an individual sample. This problem is exacerbated because both a single sample and a sampling distribution are often visualized as a histogram, and so their visual similarity can promote conceptual confusion between them (Gok & Goldstone, 2022).

Saldanha and Thompson (2002) note that a poorly performing student may have a shaky grasp of the sampling distribution’s interpretation and replace it with an interpretation that is simpler, such as a single sample. van Dijke-Droogers et al. (2021b) report similar challenges in a study with top-performing students in the Netherlands. In this study, the authors designed an activity that involved experimenting with physical chance devices and computer simulations. At the end of the activities, students still showed cross-level confusion. For example, when judging the probability of a sample mean below a certain threshold, students referred to a single sample graph instead of the sampling distribution of the means graph. When asked about the probability that an individual observation was below a certain threshold, they referred to the sampling distribution graph instead of the population graph.

A promising pedagogical approach to prevent such confusion is to distinguish sampling distribution graphs from sample and population graphs visually. van Dijke-Droogers et al. (2021a) found that visual differentiation between sample and sampling distributions helped students interpret sampling distributions accurately. In another study, Jacob and Doerr (2014) introduced a simulation activity that focused on distinguishing population, sample, and sampling distribution graphs. Post-activity, students were successfully able to predict how sampling distributions with different sample sizes would appear for a given population. Dynamic boxplot visualizations seem to be another promising way to depict sampling distributions for emphasizing their variability and its relation to statistical inference (Pfannkuch et al., 2015). Overall, the concrete and dynamic nature of these graphs may ground students’ conceptual understanding.

The confusion about variability in sampling distributions, however, seems to go beyond a simple problem of misidentification. Some studies find that students may still have difficulty predicting the shape of the sampling distribution graphs even when correctly identifying that the graph represents a distribution of sample statistics (Lehrer et al., 2014; Saldanha & Thompson, 2007; Vanhoof et al., 2007). As discussed in the previous section (Refer to 2.3.), these findings can be accounted for by the attribute-substitution model of heuristic judgment: When confronted with a computationally difficult problem, students may unconsciously and instantly substitute a simpler analog of the problem.

#### Intuitions about the law of large number: *n* (absolute sample size) versus *n*/*N* (proportional sample size)

The absolute sample size determines how closely a sample mean will match the population mean because the error in the sample estimate is inversely related to the sample size. However, several studies have indicated that students believe that it is not the absolute size of the sample (*n*) but its proportion to the population size (*n*/*N*) that predicts how closely a sample mean will approximate the population mean (Braham et al., 2013; de Vetten et al., 2018; Maxara & Biehler, 2006; Meletiou-Mavrotheris & Paparistodemou, 2015; Pfannkuch et al., 2015). Because of this belief, students often attempt to investigate a certain portion of a population during simulation activities. Even students relatively strong in statistics hold this belief.

A basic intuition for this conception seems to be that a smaller sample is a much poorer representation of a population than a large sample because of its respective proportion to the population size is small. A think-aloud protocol in Pfaankuch et al.’s study further reveals the reasoning process: “As the population gets larger, a small sample won’t reflect exactly what the data is, because in a population of a million, there will probably be more than 30 extreme values, and so if you’re taking a sample of 30 [you] could get all those 30 extreme values, which could completely sort of skew your data” (p. 353). The quote reveals that the student does not appreciate that as the number of extreme values increases in a larger population, so does the number of other values.

To target this conception, Smith (2004) developed a classroom activity with simulations in which students first varied *n*/*N* (the ratio of sample-to-population size) and kept *n* (the absolute sample size) constant, and then, vice versa. Pre- and post-tests gauged students’ reasoning through True/False responses to the statements “You need to obtain a sample that is at least 10% of the population to get a reliable estimate of the population parameter” (False) and “for large population sizes, the size of the population is irrelevant to the reliability of the sample estimate; what matters is the absolute size of the sample.” (True). Students' choices of correct statements from the pre- to post-test improved dramatically (from 7 to 53% for the first item and 0–86% for the second item).

Several studies reference this study as a successful intervention in addressing this challenging concept (e.g., Ben-zvi et al., 2015; Garfield et al., 2008). However, we are skeptical that Smith’s evaluation items have captured students’ conceptual understanding. It is not surprising that if students observe a phenomenon, then they will be able to report back what they have just seen. However, this does not mean that students have gained a mechanistic understanding of the processes involved, such that sampled data above the mean balances other sampled data below the mean, and it becomes vanishingly unlikely as the sample size increases that all the random data sampled from a population will be above or below mean. Moreover, the long-term retention of these rules is also doubtful. Research indicates that even when feedback initially modifies students' intuitive concepts, which may contradict normative rules, the original thinking patterns tend to resurface within a short period (Butler et al., 2011). We conclude that research has yet to identify convincing pedagogical approaches to address students’ intuitions about the absolute versus proportional size of the samples.

#### Challenges with inference from a single sample

Consider a question such as “Given the population distribution, how likely is it to observe that a sample of 10 data points has a mean of 50?”. The sampling distribution is directly relevant to such questions. In this case, the sampling distribution of means would show the results of randomly collecting sets of 10 data points and determining their means. If a low percentage of these means (e.g., 5%) has a value as deviant from the population mean as the observed single sample, then one can reasonably infer that it was taken from a population that systematically diverges from the population in question. While this logic is not overly complex and is foundational for statistical inference, the statistics education community has yet to find a way to convey it to students of introductory statistics reliably.

For hypothesis testing with simulations, students are often tasked with constructing hypothetical probabilistic models from which they can draw multiple samples to form a sampling distribution of statistics. Then, they determine the unusualness of certain empirical data of interest against this hypothetical population or model (see Fig. 1). Studies found that students are not able to draw statistical inferences from a single sample when they are asked to do so (Arnold et al., 2017; Braham et al., 2013; de Vetten et al., 2018; Eliason & Jones, 2020; Jacob & Doerr, 2014; Saldanha & Thompson, 2002; van Dijke-Droogers et al., 2021b). A persistent obstacle seems to be that the inferential importance of the sampling distribution is not sufficiently impressed on students who are inclined to make more direct comparisons between the observed data and the hypothesized population.

According to Saldanha and Thompson (2002), students use *representativeness heuristics* instead of constructing an internal image of the sampling distribution while performing statistical inference. In another study with simulations, van Dijke-Droogers et al. (2021a) taught students to construct sampling distributions, compare sampling distributions with different sizes, and determine the extreme values in the sampling distributions. Following this instruction, when asked to make inferences about the population distribution, students made inferences about the population distribution based on only a single sample with a small size without considering the sampling variability. Additionally, when they were asked to determine the probability of the sample mean falling below a specific value, they did not refer to the sampling distribution graph available on the screen but instead referred to the distribution of a simulated sample. Similarly, Braham et al. (2013) found that even though students had just explored the sampling distribution graphs and elaborated on their meanings, they were not ready to perform statistical inference based on a single sample. Students claimed one cannot draw inferences from only one single sample. In both studies, the students needed to be explicitly cued to use the sampling distribution graph for making the inference. Sampling distribution simulations can, indeed, create the misconception that one needs to literally take multiple empirical samples to perform valid statistical inference (Watkins et al., 2014). While simulations might aid with the process of taking multiple samples, they may be less helpful in encapsulation of the process to a single entity, which is a crucial step for statistical inference (Eliason & Jones, 2020).

Over the last decade, bootstrapping and randomization tests have been recommended as contemporary methods to introduce statistical inference concepts (e.g., Chance et al., 2016). Given that understanding the simulated distribution of statistics in randomization tests also demands a similar level of higher-order thinking as in sampling distributions, students might likely encounter similar conceptual difficulties. Indeed, empirical evidence suggests that persistent challenges remain such that students conflate the hypothetical nature of the simulated samples and an actual empirical sample (Brown, 2021). A specific area of confusion arises in bootstrapping; students regard the original sample as the entire population and thus sample only a part of it instead of performing resampling with replacement using the original sample size (McLean & Doerr, 2015). Overall, we conclude that research has yet to identify promising pedagogical approaches to facilitate students’ understanding of sampling distributions of statistics and their ability to spontaneously use simulations for statistical inference.

#### Context’s influence on reasoning

Prior research suggests that students are sensitive to the context in which sample size problems are presented whether they are trained with simulations (Abrahamson et al., 2006; Maxara & Biehler, 2006) or not (Findley & Lyford, 2019).

In one study, Maxara and Biehler (2006) tested undergraduate mathematics education students’ reasoning to assess their long-term understanding of the empirical law of large numbers and sampling distributions before and after receiving a simulation-intensive introduction to statistics course. The authors interviewed students with various story problems with a similar statistical structure. Even though each problem required normatively similar solutions, students’ responses showed little consistency across different questions. For example, for the maternity ward task (Saldanha & Thompson, 2002), they frequently used the law of large numbers justification, whereas they invoked the growing possibilities heuristic on an analogous scenario about reaching a passing grade on an exam with just guessing (e.g., “I have more opportunity to guess correctly with more questions.”). On two other analogous questions about the chances of winning a game in a casino and predicting elections from a survey, the students were explicitly cued for analogies between the two (“Can you see analogies between the tasks?”). Even though this cueing helped students apply the correct solution from one task to the other, it did not improve the quality of their explanations of the correct answer. Overall, the authors reached two conclusions. First, students’ contextual familiarity may either facilitate or sidetrack their statistical reasoning on story problems. Second, problems constructed regarding statistics (such as survey tasks) are easier to solve than probabilities (such as slot machines).

Findley and Lyford’s (2019) study support Maxara and Biehler’s conclusion that contextual familiarity influences students’ reasoning about story problems. However, it challenges the conclusion that probabilistically presented contexts are necessarily more difficult. Findley and Lyford asked students to draw graphs across two different story problems, one constructed as the distribution of a range of average pennies in circulation by their production years and the other as the averages of multiple dice rolling. In both tasks, students constructed the distribution of averages for a sample size of 2 and then 10. More students responded to the dice task correctly than the penny task. On the dice task, they correctly predicted that the average should cluster closely around the middle of the range with a larger sample size. However, for the penny task, they incorrectly expected that a larger sample should produce a wider range of averages (growing possibilities heuristics). The authors concluded that students’ daily life knowledge of dice freed them to focus their reasoning on the average that these samples would produce while the unfamiliar context of the penny task caused them to dissipate their focus and led to a less useful reasoning path.

After a review of these two studies, it is difficult to make predictions regarding how the specific aspects of story problems will influence students’ reasoning because researchers compared stories that differed along various dimensions from each other without isolating particular aspects. Nevertheless, it seems that students’ piecemeal contextual knowledge may suppress the information conveyed by the statistical models with which they are trained.

As a promising approach, van Dijke-Droogers et al. (2021a) found that using the same simulation software to model various scenarios can support extracting context-independent structures of the tasks. The authors found that this approach shifted students’ understanding from context-specific interpretations toward more abstract, higher-level statistical reasoning. However, it has yet to be shown if students can demonstrate broader transfer effects without the presence of the simulations.

### Discussion: a grounded cognition perspective to sampling simulations

In the second section, we reviewed educational studies that teach students statistical sampling with computer simulations. The findings suggested tentative benefits of computer simulations in terms of building general statistical reasoning, skills, and habits of mind over time. When fundamental concepts were more specifically investigated, however, distinguishable patterns were present which point to persistent challenges. Among those, first, was students’ focus on the local changes in graphs at the expense of global patterns which hindered their conceptualization of the law of large numbers. Second, when the activities moved from lower-level (e.g., single sample distributions) to higher-order graphs (e.g., the collection of statistics), students were at risk of losing their grasp of what the graphs represent. Third, students often showed a lack of a process-based understanding (that is, understanding how) of the principles (e.g., the law of large numbers). Fourth, when left to their own devices, students were not able to apply what they had learned in conducting simulations to make inferences about a single sample. Fifth, students’ problem–solution approaches showed inconsistency across different contexts that could have been organized under the same statistical principle. A quick judgment of the summary of these results may lead us to conclude that students, with and after simulations, display patterns of reasoning in ways that would be expected without any training. That is, they are easily swayed by piecemeal contextual knowledge without a causal mechanical understanding of probabilistic processes, and simulations sometimes even cause additional misconceptions because of learners’ incorrect interpretations of graphs.

It should be noted that the educational psychology research community seems to broadly agree that causal claims about whether an instructional intervention has benefits on learning should be based on randomized experiments (e.g., Brady et al., 2023; Grosz, 2023; Mayer, 2023). Correlational and qualitative studies, on the other hand, can provide grounds for generating hypotheses to be tested in future experimental research (Brady et al., 2023). To this end, we interpret our review’s results in light of a broader grounded cognition perspective.

#### Bidirectional relationships between perception and cognition

Grounded cognition posits a bidirectional relationship between lower-level perception and higher-level conceptual thought. In one direction, perceptual features that co-occur in the environment are linked through associative learning, forming concepts through indirect associations once the direct associations become automatic (Sagi & Tanne, 1994). This perspective helps explain our findings that repeated exposure to simulations enhances students' overall understanding of statistics concepts (see “Benefits of simulations for general statistical reasoning abilities and skills” section). In the other direction, perceptual processing is strategically adapted to support cognition (Goldstone & Barsalou, 1998). For example, to achieve conceptual goals, irrelevant perceptual features of objects are de-emphasized, while relevant features are accentuated (Goldstone, 2019). Such a mechanism could be involved in our findings that children, not having yet formed normative conceptual goals in statistics, tend to focus on local data aspects at the expense of recognizing global trends critical for data investigations (see “Local versus global view of data” section).

An important pedagogical implication of this bidirectional link between perception and conception is to enable students’ repeated exposure to simulations with activities targeting normative conceptual goals in mind. Such a perspective expands the role of simulations beyond mere introductory demonstration tools for beginners. Instead, it suggests that simulations are better situated as tools to think with as students increasingly build more sophisticated concepts. For example, as they advance their understanding of statistical concepts, students may more easily gain a more distributional view of sample data and an object-oriented understanding of sampling distribution graphs. Studies suggest that as people gain expertise, they become more skillful at extracting relevant information and recognizing complex patterns from objects (Kellman & Massey, 2013; Yu et al., 2018a, 2018b). However, adaptation of perception is slow, as befits perception’s early position in information processing (Goldstone, 2019). It is appropriate that perceptual processes change conservatively given that their outputs serve as the inputs for all downstream processes in the flow of information processing. Based on this notion, an open empirical question emerges: Whether prolonged exposure and experience can mitigate the reported challenges.

Additionally, quasi-experimental studies have provided some evidence that the benefits of sampling simulations go beyond the specific learning scenarios in which they were situated. This includes the development of general statistical habits and norms (van Dijke-Droogers et al., 2021b) and a broader understanding of statistical concepts (Hancock & Rummerfield, 2020). Research in other scientific fields has shown that dynamic and spatial simulations help learners implicitly construct mental models, which they can flexibly and spontaneously apply to future, superficially dissimilar tasks (Day & Goldstone, 2011). These findings collectively suggest tentative benefits of simulations in facilitating the transfer of learning, though more direct evidence is needed, especially in the context of statistical sampling.

#### Grounded simulation designs

**Fig. 5:**
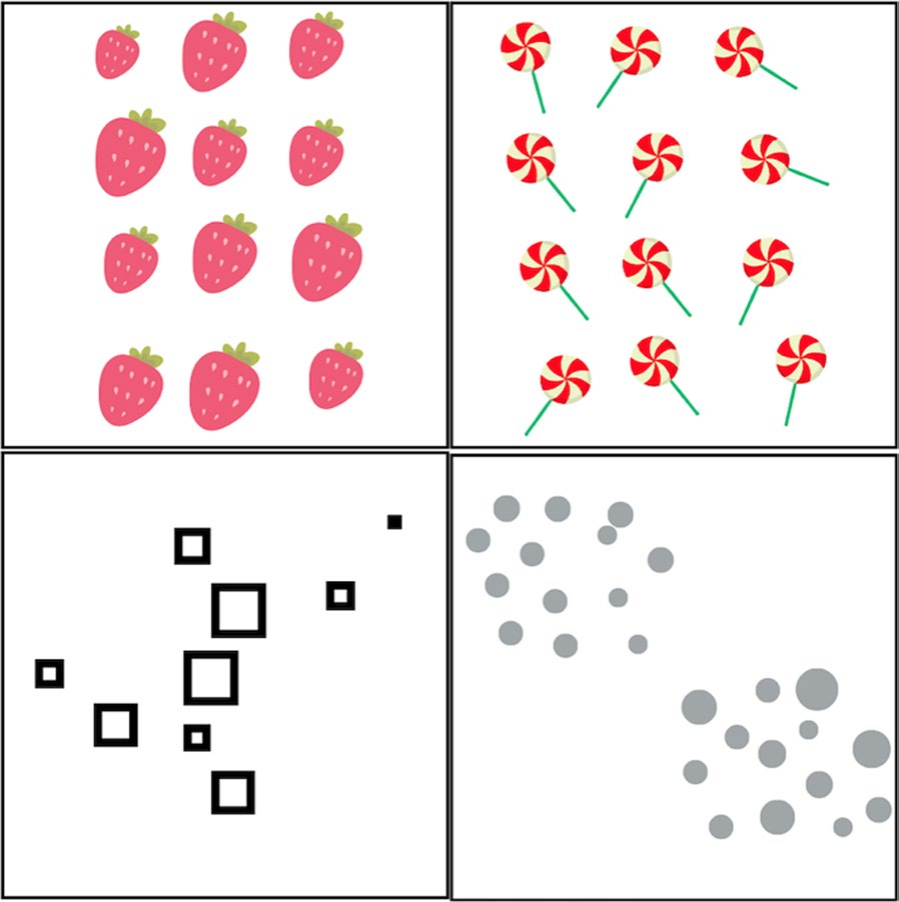
Sample stimuli from the ensemble perception literature. *Note.* Examples of object groups from which people were found to efficiently discern statistical summaries without explicit enumeration: mean and variance of the size of strawberries (Yang et al., 2018), mean and variance of the orientation of lollypops (Yang et al., 2018), the centroid of squares (Rodriguez-Cintron et al., 2019), and the mean size of dots (Chong & Treisman, 2005). The figures are redrawn from their original sources

Controlled laboratory experiments have provided evidence that people can easily discern statistical summaries of objects that vary along a particular visual or spatial dimension. This ability extends across a diverse range of dimensions and objects. For example, people can estimate the average emotional expression in a collection of face images ranging from happy to sad (Elias et al., 2017; Haberman & Whitney, 2009). Similarly, they can determine the average luminance of dots, the size of squares (Rodriguez-Cintron et al., 2019), circles (Chong & Treisman, 2005; Lau & Brady, 2018), and strawberries (Yang et al., 2018). This ability also applies to estimating the length of lines (Bauer, 2017), the orientation of lollipops (Yang et al., 2018), and even to more abstract attributes, such as the lifelikeness of objects (Leib et al., 2015) (see Fig. 5). These objects are clustered onscreen and centered around a focal point. The participants are then asked to estimate a statistical summary, such as the average, variance, or centroid of the objects that differ along one dimension. They may be asked to position a slider to reflect their visual estimation or verbally compare two sets of objects, such as which one has a larger or smaller mean or variance.

Notably, these estimations, referred to as ensemble perception, are made without any explicit calculation or detailed encoding of each individual item displayed (for a review, see, Cui & Liu, 2021). The global statistical impressions are formed rapidly and early in vision. Sensory neurons quickly adjust to the statistical properties of the visual input, and from just a few glances, people can create a general representation of a scene (Chong & Treisman, 2003). This suggests that our sensory system can extract essential statistical information from our environments without conscious effort.

Given the evidence on the close relationship between perception and conception and robust findings from ensemble perception literature, it may seem contradictory that our reviewed findings hint that students struggle to interpret the statistical nature of pedagogical simulations even with deliberate efforts of the educators. Cui and Liu (2021) offer an explanation for this disconnect. First, particular graphs used in statistics education, such as histograms, introduce perceptual difficulties. For example, what the axes represent is often counterintuitive (Kaplan et al., 2014; Lee & Meletiou-Mavrotheris, 2003), and students sometimes treat a histogram’s bars as distinct objects (Newman & Scholl, 2012). Second, educational tasks typically bear additional cognitive demands, such as transforming the estimated visual summaries into numerical values. This suggests that students face challenges at two levels: perceptual understanding of the graphical representations and cognitively transforming that information into numerical values.

**Fig. 6:**
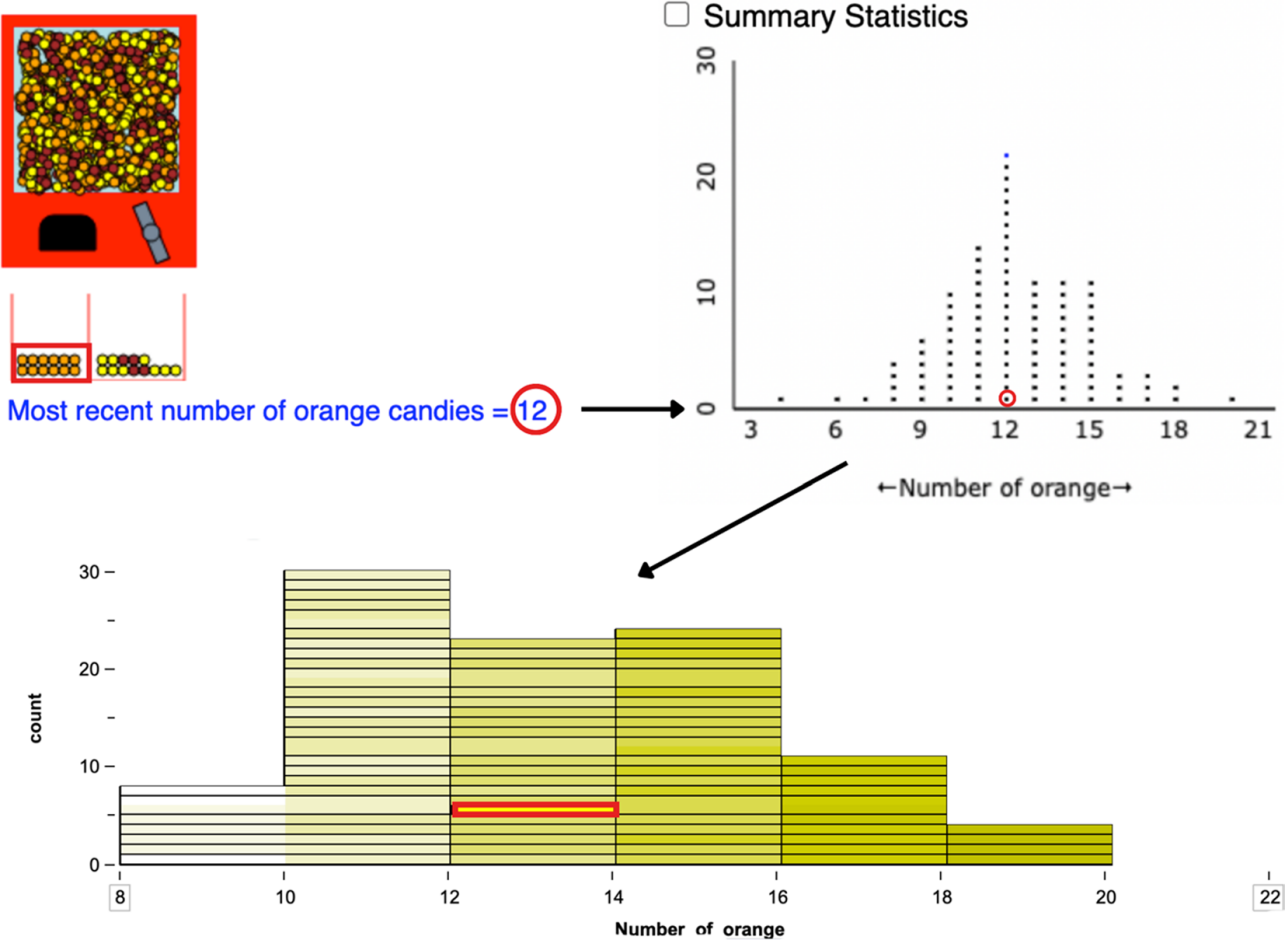
Grounding examples in the sampling simulations. *Note.* Some practical examples of grounding are animations that depict the random selection process and the transition from iconically depicted situations to dot plots to histograms. The screenshots are from the Rossman and Chance applet (top panel) and TinkerPlots (bottom panel)

Adopting a grounded cognition approach may enhance the effectiveness of simulations at both levels. That is, simulations can be designed to combine abstract concepts and statistical sampling mechanisms with concrete and familiar referents akin to daily experiences to facilitate students’ meaning making. Indeed, several of the reviewed studies already take advantage of this notion (see Fig. 6). For example, by default, Tinkerplots and Fathom, the most frequently used software programs for teaching statistical reasoning, use dot plots instead of histograms. In addition, Tinkerplot uses animation to transition between display types, explicitly cueing the relationship between different representations. Moreover, animations that depict random selection processes are found in software like the Rossman and Chance applet, Tinkerplots, and the Virtual Rice Lab. Nevertheless, these could be further grounded to better mirror the tangible experiences and contexts familiar to students.

For example, in a study by Yu et al., (2018a, 2018b), the researchers designed an animation that illustrated two machines in a factory producing balls under different settings. Going beyond identical-looking dots in a dot plot and bars in a standard histogram, the authors used tokens that resembled the actual objects (balls) representing each observation. For the axes, no numeric tags were used to indicate the value of the variables. Instead, the Y axis represented the actual height reached by the bouncing balls, and the individual differences between the balls were represented by systematic variation of their colors on the X axis. Thus, the data measurements were directly shown on a naturally corresponding visual dimension, which avoided the necessity to calculate the variables' values explicitly or to map the dimensions represented by the axes effortfully.

In a recent controlled experiment, we tested the promises of this grounded approach for the design of sampling simulations (Gok et al., 2024). We used tokens to represent the individual data points to ease the representational competence required for grasping histograms. We physically distinguished the tokens for collected statistics from singular observations to avoid confusion. Additionally, we animated the aggregation process of averaging when constructing the collection of means so that students could construct a spatial representation corresponding to the informal notion that “low and high scores are more likely to cancel each other out with larger samples.” Compared to a standard histogram simulation, our initial results suggest that grounding with concrete simulation helped students better understand the situation during their inquiry activities, but it did not show more advantageous transfer effects at the post-test.

It has been shown elsewhere that concrete representations can limit the transfer of knowledge to situations having different concrete manifestations (Goldstone & Sakamoto, 2003). Applying these results to sampling, students who learn sampling processes through tangible materials or token-based graphs might not be expected to apply their knowledge when encountering generic histograms in a textbook later. However, this possibility does not mean that concrete representations should be avoided for better generalization of learning. A more promising approach, referred to as concreteness fading, is to start with concrete representations and gradually fade them to more abstract ones so that students can benefit from a grounded approach while generalizing what they have learned (Fyfe et al., 2014).

Consistent with the concreteness fading approach, another route for the grounded cognition approach in pedagogical simulations is to employ tangible objects before students transition to computer simulations. Examples of such objects have included marbles for drawing samples from a box (Abrahamson, 2014, van Dijke-Drookers et al., 2021b), different-colored candies to demonstrate sampling distributions (Hancock & Rummerfield, 2020), a video demonstration of an instructor manually shuffling data on paper to teach the randomization test function in R (Zhang et al., 2022), and data cards in a population bag (Arnold et al., 2017). Although many studies did not evaluate the particular impact of these tangibles on learning, Hancock and Rummerhield’s quasi-experiment revealed that the group that engaged with tangible activities before computer simulations had significantly higher improvement in their exam scores compared to the group that engaged with only computer simulations. Notably, the improvement was not for the items that specifically gauged sampling distribution knowledge but in the overall understanding of statistical concepts. Zhang et al.’s (2022) controlled experiment yielded similar results. Students who watched a hands-on video before using R simulations demonstrated better understanding than those who used R simulations alone. These results underscore the educational benefits of concrete precursors, whether through observation or direct experience.

#### Guiding simulation explorations: the roles of visual routines and reification

While we have recognized grounded simulation design as a promising avenue for future direction, guiding students’ perceptual engagement with graphs is likely at least as important. Evidence suggests a strong link between perceptual patterns and graph interpretations. For instance, when analyzing histograms to estimate the mean, students who employ an incorrect strategy show a tendency for horizontal eye movements, indicating they treat each bar as a separate case, whereas students using a correct strategy exhibit vertical gaze patterns (Lyford & Boels, 2022). A local perspective on the data correlates with more fixations on individual points, whereas those with a global perspective demonstrate longer movements across the histogram (Schreiter & Vogel, 2023). In addition, experts allocate more time to textual elements providing context, such as titles, legends, and axis labels, which novices tend to overlook (Harsh et al., 2019).

**Fig. 7:**
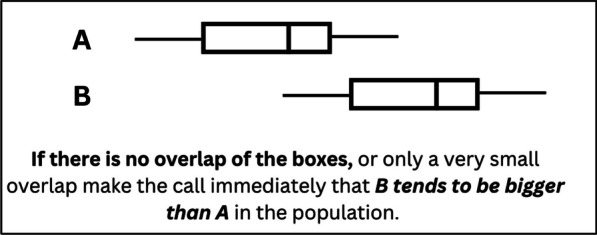
Checklist example. *Note.* Checklists guide visual routines while students interpret simulations. The figure is redrawn from Arnold et al. (2017)

Complementing this relationship between graph perception and interpretation, two reviewed findings highlighted the importance of checklists for improving students’ interpretation of simulations. These checklists range from intangible norms, like structured inquiry processes (Makar et al., 2011), to tangible guidance materials that help interpret variability patterns in graphs (Arnold et al., 2017, see Fig. 7). Simulations are perceptually ambiguous. They are typically complex and have numerous components which could be attended to or grouped into structures. Whether through physical tools or established norms and practices, checklists graft interpretive organization onto a rich simulation's blooming and buzzing confusion.

Correspondingly, Yu et al., (2018a, 2018b) found that inquiry through grounded simulations improved learning outcomes when paired with analytical feedback that explained pattern changes but not with simple right-or-wrong feedback. These results are congruent with our understanding of the visual system’s functioning, where rapid and intuitive visual processing effectively picks up basic statistics but falls short of a more sophisticated understanding of graphs, such as the relationships among the values or the comparison between different groups, which requires the visual system to isolate relevant parts of the graph across time slowly and serially (Xiong et al., 2022). The visual system relies on 'visual routines'—a series of operations like focusing attention, indexing, and activation spreading—to extract complex properties and relations from visuals (Ullman, 1987). These routines, crucial for extracting sophisticated data relations, often require guidance to form effective perceptual habits for graph analysis (Goldstone & Wilensky, 2008). Teaching students these visual routines, with relevant eye movement guidance, can enhance learning from graphs (Michal & Franconeri, 2017; Michal et al., 2016). Verbal instruction can change the values of a graph that people attend over time (Michal et al., 2016). Visual attention is more efficiently allocated to targets when they are explicitly labeled (Lupyan, 2017), and labels can even help objects be seen that would otherwise be missed (Lupyan & Ward, 2013). Such labels also possess object-like properties that aid in compressing information and representing rich associative connections while filtering complex and ambiguous perceptual experiences in the service of conceptual goals (Son et al., 2010).

Similarly, statistical notions also adopt object-like properties. Abstract notions typically begin as actions and processes, gradually condense, and finally, the learner conceives of the notion as an object. This stage, referred to as reification (Sfard, 1991), is an ontological shift where a complex pattern or set of relations is viewed as an object in its own right. Reification allows grasping large amounts of data from several processes at one glance (Eliason & Jones, 2020; Font et al., 2013; Sfard, 1992). An important finding from the reviewed studies was that simulations may help foster an understanding of statistical processes, such as how sampling distributions are constructed by taking means of all samples from the population. However, simulations fell short in promoting the reification of these processes, such as viewing the sampling distribution as a theoretical and abstract object. Yet, reification is the building block for more advanced mathematical constructs and processes, such as calculating the likelihood of observing certain values or making inferences from a single sample based on the sampling distribution.

An argument in the reviewed literature has been that expressing statistical notions as objects in language (e.g., referring to “the spread” instead of saying “the dots are spread out”) indicates reification of the notion (Bakker, 2004). Eliason and Jones (2020) argued for the benefits of explicit instruction regarding theoretical sampling distributions for reification. We propose that visual routines could also be instrumental in promoting such reification, enabling students to visualize statistical concepts as tangible objects with discernible attributes and properties. Sophisticated visual processes such as figure-ground separation, marking, following, and annotating, typically employed in object perception, can be adapted for abstract concepts, transforming these notions into visual objects within students' understanding.

#### Summary: pedagogical proposals for future empirical testing

**Fig. 8:**
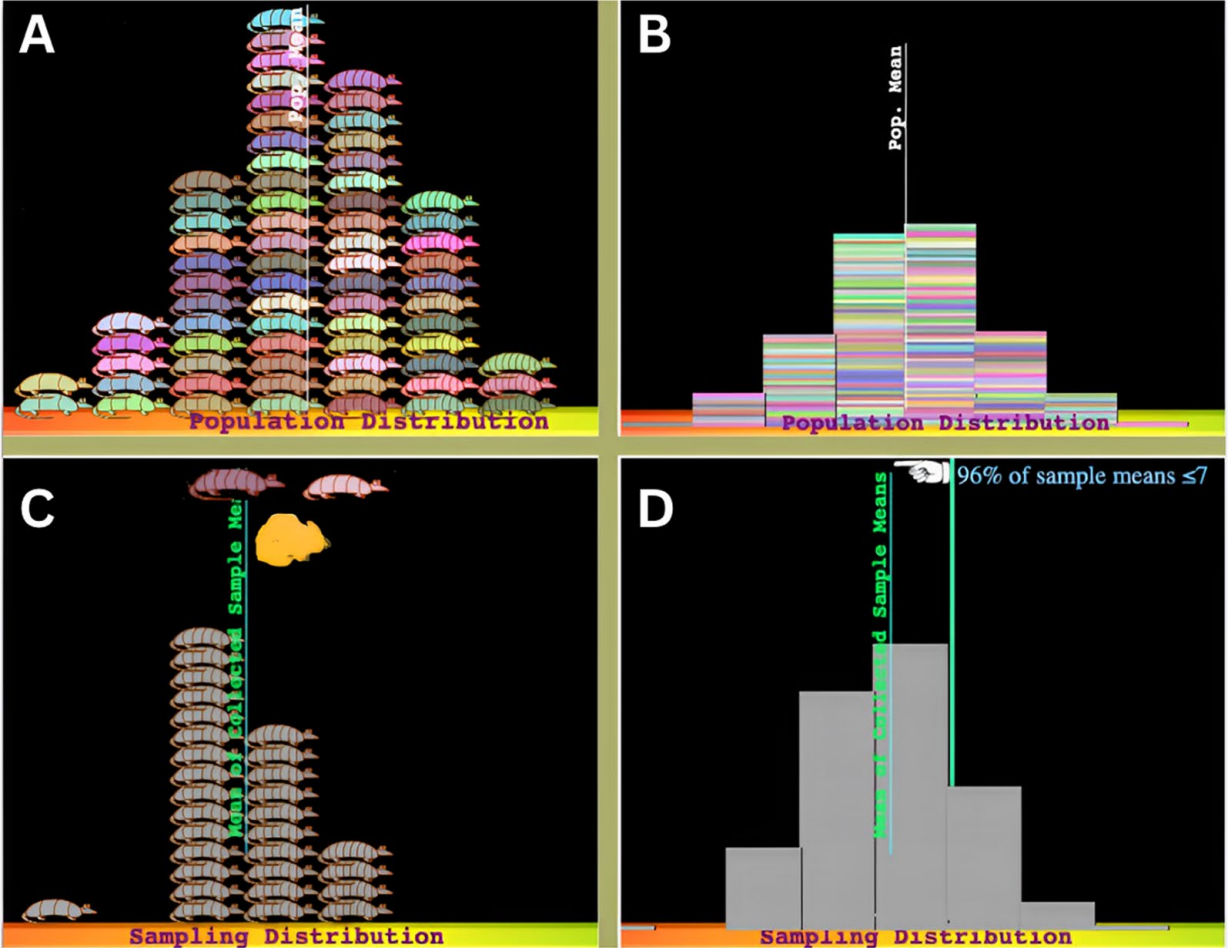
An empirical investigation of our proposals. *Note.* The figure showcases an application of our proposals, as investigated in our ongoing empirical studies. **A** The default option of the simulation is a token-based graph so as to ease the representational competence required to understand the distribution. **B** The graph is transformed into an idealized form to facilitate a more generalized comprehension of the graphs. **C** The collected means are represented with icons different from those representing singular observations to avoid confusion. Additionally, the averaging process is visualized, creating a spatial understanding that mirrors the concept of the mean as a balance point. **D** The sampling distribution graph is idealized as a stable object (reified), and the rejection region is marked for guiding perceptual routines to the graph’s important properties crucial for conducting statistical inference

Finally, we outline the pedagogical insights that have emerged from the review. Note that the following points are not proposed as definitive guidelines. Rather, they are dimensions we regard as meriting close attention in future empirical research and instructional design (see our own efforts in Fig. 8). We call for future controlled experiments to refine these insights through incremental testing along these dimensions.

- Students build habits of mind through repeated exposure to simulations.

Percepts and concepts are interlinked and shaped in a mutual, recurrent, and bidirectional manner. Students’ past experiences guide their interpretations of the simulations, and they iteratively refine their interpretative skills based on these observations. The frequency with which students are provided opportunities to engage with simulations is a crucial pedagogical dimension. Simulations serve not merely as an entry point for novices but as potentially effective tools to think with as students’ progress in their statistical training.

- Familiar, intuitive, dynamic representations ground students’ meaning making from simulations. Idealized representations generalize learning.

Traditional statistics diagrams are often confusing for novices as the meaning of their physical and spatial properties mismatch with students’ prior experiences with these properties. These simulations can initially be grounded in familiar tangibles, token-based graphs that visually resemble the data they represent, animations that translate statistical processes into dynamic spatial representations, and visual cues that mirror the underlying conceptual similarities and distinctions. These grounded simulations should gradually transition to more idealized forms for fostering transfer of learning to different situations and facilitating adept use of standard statistical tools.

- Visual routines need to be guided during inquiries using simulations.

Statistical simulations inherently possess perceptual ambiguities, making it challenging for novices to direct their attention to diagnostic elements. This can lead students to develop ineffective perceptual habits. Previous research indicates labels, verbal instructions, checklists, and visual cues may be effective methods for directing students’ attention. Another solution may be a more socially grounded approach, where students view simulations with their teachers. The gestures and cues provided by the teacher can guide students’ routines.

- Simulations and verbal materials have separate affordances.

Due to the implicit nature of perceptual learning processes, students may often be unable to verbalize their learning from the simulations. Linguistic materials and verbal instructions can turn intuitive and implicit learning gained from the simulations into explicit and verbalizable ideas.

- Statistical processes depicted in the simulations should be reified as foundations of more advanced concepts and practices.

Merely exposing students to statistical processes through simulations does not guarantee their reification of these processes. Yet, reification is crucial for building blocks of more advanced concepts and practices. After engaging in inquiry activities using simulations, the investigated processes should be reified through explicit instruction on theoretical principles and guidance on visual routines that allow grasping complex processes at one glance.

## Conclusion

Many view statistics as a discipline that is not intuitively graspable. Students in statistics classes are frequently advised to set aside their intuitions to avoid mistakes and adhere to the mathematical proofs they were taught instead. This perspective is memorably expressed by John Von Neumann: “In mathematics, you don't understand things. You just get used to them." Historically, humans’ intuitive and experiential learning systems were believed to interfere with the complex, abstract, and rule-based system of thinking required in fields like science and mathematics. These two systems have been thought to produce incompatible solutions to problems.

In this review paper, we explored the promises of an alternative view that emphasizes the interaction between Systems 1 and 2. We proposed that perception–action routines are built to support formal reasoning, and formal reasoning is simultaneously built out of trained perception–action routines. This perspective repeatedly appeared to us as illuminating when we reviewed the educational literature on statistical sampling simulations. We do not claim this perspective will always reach desirable solutions and acknowledge that it does not exhaust what the reviewed papers have to offer. However, we hope this grounded cognition perspective will be an important theoretical addition to inform the pedagogical methods for teaching difficult concepts such as statistical inference.

## Data Availability

Not applicable.

## Footnotes

1 The works we cited by Kahneman and Tversky and their follow-ups do not distinguish between intuitive judgments versus heuristics, and we also used the two terms interchangeably here. However, Evans (2012) distinguishes between them because intuitive judgments are based on feelings and a wide range of typically unconscious contextual cues, whereas heuristic judgments are simple rules based on few cues and partial information.

2 The correct answer is the smaller hospital.

3 Note that there are 365 samples for each hospital. The collection of proportion of boys for each sample (day) forms an empirical sampling distribution. The correct answer is still the small hospital (Sedlmeier, 1999).

4 Grasping sampling distributions of statistics requires turning a statistical process (taking statistics of samples) into a mathematical object that can be reasoned with, and it is hard to transition from a one-time process (e.g., taking a single sample mean) to an abstract entity (a sampling distribution of means) created by repeatedly applying the process (Cobb, 2007).

5 Note that this explanation accounts for people’s incorrect predictions that a sampling distributions’ standard deviation will not change with sample size. The incorrect prediction that it will get larger with larger sample sizes can be accounted for by the belief that *everything gets larger with bigger sample sizes* (Watkins et al., 2014).

6 cf. review, Evans (2008) for the counterargument from the evolutionary perspective which attributes the advantage of frequency format to System 1 operations. Accordingly, humans have evolved a cognitive module for processing frequency information in the environment without requiring a difficult calculation.
